# Endothelial ATP-Sensitive Potassium Channel Protects Against the Development of Hypertension and Atherosclerosis

**DOI:** 10.1161/HYPERTENSIONAHA.120.15355

**Published:** 2020-07-13

**Authors:** Yiwen Li, Qadeer Aziz, Naomi Anderson, Leona Ojake, Andrew Tinker

**Affiliations:** The Heart Centre, William Harvey Research Institute, Barts and the London School of Medicine and Dentistry, Queen Mary University of London, United Kingdom.

**Keywords:** atherosclerosis, endothelium, hypertension, ion channels

## Abstract

In the endothelium, ATP-sensitive potassium (K_ATP_) channels are thought to couple cellular metabolism with membrane excitability, calcium entry, and endothelial mediator release. We hypothesized that endothelial K_ATP_ channels have a broad role protecting against high blood pressure and atherosclerosis. Endothelial-specific Kir6.1 KO mice (eKO) and eKO mice on an apolipoprotein E KO background were generated (A-eKO) to investigate the role of K_ATP_ channels in the endothelium. Basal blood pressure was not elevated in eKO mice. However, when challenged with a high-salt diet and the eNOS inhibitor L-NAME, eKO mice became more hypertensive than their littermate controls. In aorta, NO release at least partly contributes to the endothelium-dependent vasorelaxation induced by pinacidil. In A-eKO mice atherosclerotic plaque density was significantly greater than in their littermate controls when challenged with a high-fat diet, particularly in the aortic arch region. Levels of endothelial dysfunction markers were higher in eKO compared with WT mice; however, these were not significant for A-eKO mice compared with their littermate controls. Furthermore, decreased vascular reactivity was observed in the mesenteric arteries of A-eKO mice, but not in aorta when on a high-fat diet. Our data support a role for endothelial Kir6.1-containing K_ATP_ channels in the endothelial protection against environmental stressors: the maintenance of blood pressure homeostasis in response to high salt and endothelial integrity when challenged with a high-fat diet.

## Abstract 2

Supplemental Digital Content is available in the text.

ATP-sensitive potassium channels (K_ATP_) channels are inwardly rectifying K^+^ channels activated by declining ATP or increasing ADP levels, thus regulating a range of biological activities by linking cellular metabolism with membrane excitability.^1^ K_ATP_ channels are ubiquitously expressed in the body and are particularly prominent in the cardiovascular system including heart and smooth muscles where they are involved in a range of physiological mechanisms such as the regulation of vascular tone and blood flow.^2–5^ At the molecular level, K_ATP_ channels are composed of 4 pore-forming Kir6.x subunits (Kir6.1 or Kir6.2) and 4 regulatory sulphonylurea receptor (SUR1, SURA, or SUR2B) subunits to form a functional hetero-octameric complex.^1,6^ Tissue-specific combinations of a particular SUR with a specific Kir6.x subunit generate channels with distinct pharmacological and electrophysiological properties.

The importance of K_ATP_ channels in cardiovascular physiology has been investigated using KO mouse models.^1,7^ For example, mice with global genetic deletion of Kir6.1 and SUR2 are hypertensive and are prone to sudden death probably as a result of coronary artery spasm.^8–10^ Mice with conditional Kir6.1 deletion in vascular smooth muscle (VSM) are also hypertensive, but they did not fully recapitulate the global KO phenotype suggesting the involvement of other cellular populations of K_ATP_ channels.^2^ It is known that K^+^ currents can influence endothelial function by hyperpolarizing the cell, promoting Ca^2+^ entry and thus leading to the release of vasoactive mediators.^11^ K_ATP_ channels have also been shown to be expressed in vascular endothelial cells (ECs) and Kir6.1 (and possibly Kir6.2) is thought to be the underlying pore-forming subunit for this channel.^12–15^ Using cre-loxP technology, we conditionally deleted the Kir6.1 subunit in vascular ECs and showed that K_ATP_ currents were abolished in ECs.^5^ Furthermore, Ca^2+^ entry into ECs in response to K_ATP_ channel activation was also eliminated and the response of the coronary circulation to hypoxia was compromised.^5^

It is clear from our work and that of others that K_ATP_ is expressed in endothelium and that it plays an important functional role in response to stress. Furthermore, there is increasing interest in endothelial cell calcium signaling and metabolism and how they might regulate key processes such as angiogenesis.^16,17^ However, the broader role that the K_ATP_ channel may play in the circulation outside of the coronary vasculature remains to be investigated.^5^ Thus, in this study, we aimed to address the role of Kir6.1-containing K_ATP_ channels in the endothelium of conduit and resistance vascular beds, and to investigate how these channels affect vascular function at rest and in response to pathological challenges.

## Methods

The data that support the findings of this study are available from the corresponding author upon reasonable request.

All experiments were conducted in accordance with the Guide for the Care and Use of Laboratory Animals published by the British Home Office regulations (covered by project licence PE9055EAD) and by the US National Institutes of Health (NIH Publication No. 85-23, revised 1996).

### Generation of Endothelial Kir6.1 Mouse Strains

Details are given in the Data Supplement.

### Blood Pressure Telemetry

Blood Pressure (BP) was measured using implanted radio-telemetry probes PAC-10 (Data Sciences International) as described previously.^2^ Briefly, mice were anesthetized with isoflurane, 5% for initial induction and 1% to 1.5% for maintenance during surgery. The left carotid artery was isolated and the probe catheter inserted to a depth of ≈1 cm and secured with sutures. The probe body was placed subcutaneously on the left side of the abdomen. Two weeks post-surgery, continuous recordings were commenced using the Acquisition module of the Dataquest software (Data Sciences International, United Kingdom) at a sampling rate of 2 kHz for 24 to 48 hours and analyzed using Ponemah P3 plus analysis software (Data Sciences International, United Kingdom). Mice were further interrogated with a high-salt diet (8% NaCI, Envigo TD92012) and L-NAME in the drinking water (5 mg/10 mL) for 4 weeks. Twenty-four hour recordings were taken at 0, 7, 14, 21, and 28 days. At the end of the study, mice were sacrificed and the hearts harvested and weighed.

See Data Supplement for details on genotyping, organ bath and wire myography experiments, tail cuff BP measurements, analysis of atherosclerotic plaque formation, ELISA, and data analysis.

## Results

### Endothelial K_ATP_ Channels Are Involved in the Regulation of Blood Pressure in Mice When challenged With a High Salt Diet and L-NAME

**Figure 1.**
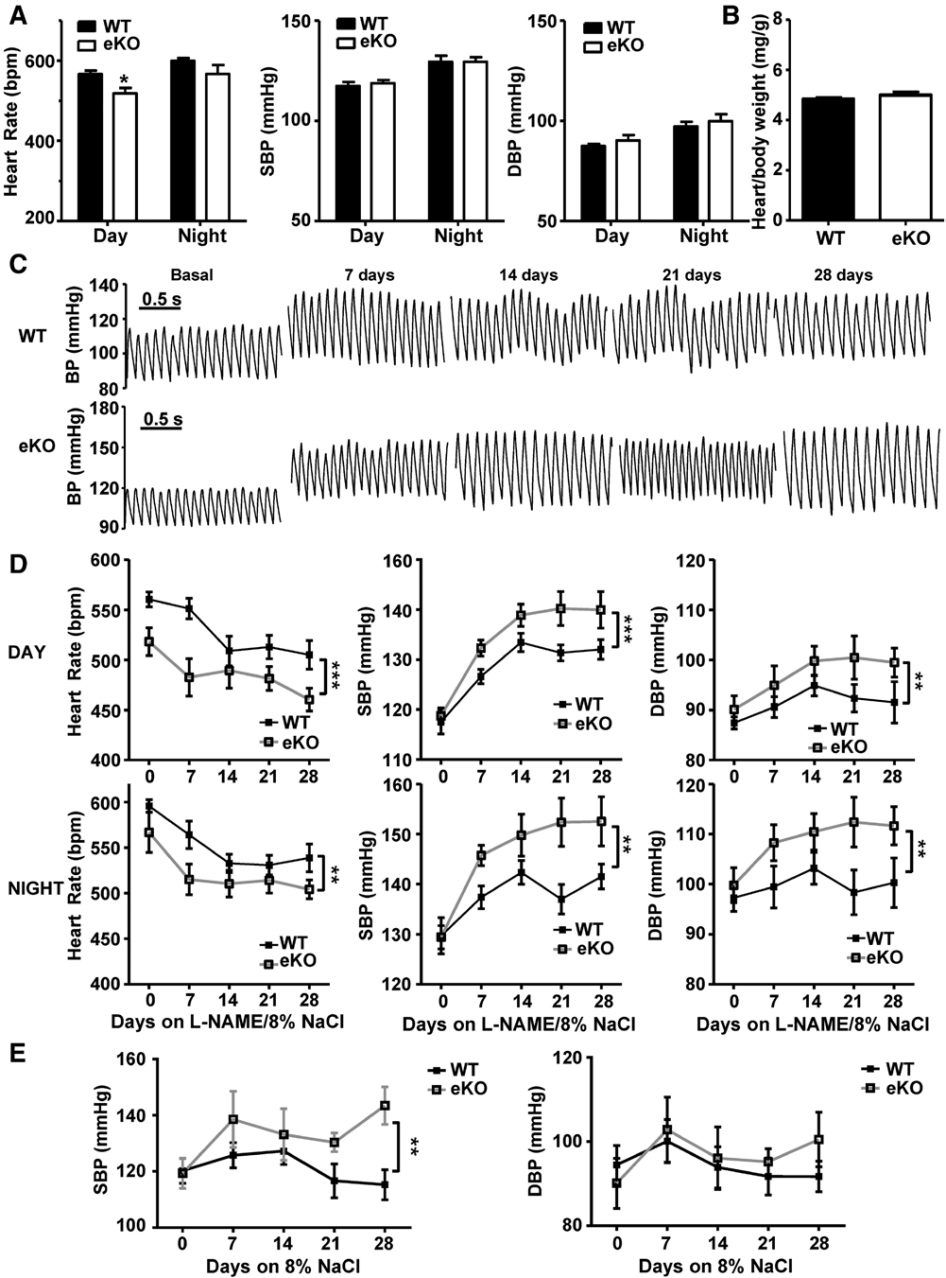
Deletion of Kir6.1 in the endothelium only affects systemic blood pressure when challenged with a hypertensive stimulus. **A**, Mean basal heart rate, systolic blood pressure (SBP) and diastolic blood pressure (DBP) measurements during 12 h light/dark cycles over 48 h. **B**, Mean heart/body weight ratio in WT and eKO mice following 28 d of L-NAME and high salt (HS) diet. **C**, Representative BP traces from a WT and eKO mouse at baseline and following 7, 14, 21, and 28 d of L-NAME and HS diet. **D**, Mean day/night heart rate, SB and DBP measurements from WT and eKO mice with diets supplemented with L-NAME and 8% NaCI. **E**, Mean SBP and DBP measurements from WT and eKO mice at baseline and following 7, 14, 21, and 28 d of only HS diet. Measurements were taken continuously over a 24 h period every 7 d for (**A–D**) and were taken at the same hour of the day every 7 d for (**E**). Data are shown as mean±SEM, n=5–6 mice, *P<0.05, **P<0.01, ***P<0.001 compared with WT.

The absence of Kir6.1 in VSM leads to hypertensive mice, but the hypertension is not as pronounced as in mice with global deletion of Kir6.1.^2^ To examine the possible contribution of endothelial Kir6.1 in BP regulation, we used continuous telemetric monitoring in conscious 8- to 12-week-old wildtype and eKO mice. Wildtype and eKO mice showed typical circadian variation in basal heart rate, systolic and diastolic blood pressure (SBP and DBP) with higher heart rate and BP at night than during the day when mice are active (Figure 1A; *P*<0.01). The basal prediet heart rate was lower in eKO mice (day: 518.1±13.86 bpm, night: 566.8±22.1 bpm) compared with wildtype (day: 560.3±7.45 bpm, night: 595.7±6.95 bpm) mice, significantly so during the day (*P*<0.05). There was no difference in prediet SBP and DBP between wildtype (day: SBP/DBP, 117.5/87.4±2.4/1.2 mm Hg, night: SBP/DBP, 129.7/97.3±3.6/2.7 mm Hg) and eKO (Day: SBP/DBP, 118.7/90.1±1.6/2.7 mm Hg, night: SBP/DBP, 129.4/99.8±2.3/3.5 mm Hg) mice suggesting K_ATP_ channels in the endothelium do not contribute significantly to basal BP regulation in mice (n=5–6, *P*>0.05). Analysis of the heart weight; body weight ratio did not reveal cardiac hypertrophy at this time point in either wildtype or eKO mice (Figure 1B). To investigate if endothelial K_ATP_ channels containing Kir6.1 play more of a role in BP regulation when challenged, we put both groups of mice on a diet with L-NAME (5 mg/10 mL of drinking water) and a high salt (HS) chow (8% NaCI) over a period of 4 weeks. Both groups became hypertensive within the first week on the diet with a decreased heart rate and increased SBP (Figure 1D, *P*<0.01). Pulse pressure was also increased in both groups. All parameters reached a relative plateau at 14 days. Significantly, the effect on heart rate and BP was greater in mice lacking endothelial Kir6.1 (Figure 1D; *P*<0.01). We also used a noninvasive tail cuff method to measure BP when mice were on a HS only diet (without L-NAME). Basal BP levels were similar in wildtype and eKO mice (*P*>0.05). After 7 days on the HS diet, both genotypes became hypertensive. In wildtype mice, SBP and DBP returned to baseline levels by day 21; however, in eKO mice, BP was significantly (specifically SBP) higher than baseline and wildtype mice at 28 days (Figure 1E; SBP WT, 119.52±4.79 mm Hg, eKO, 140.20±7.70 mm Hg; *P*<0.05).

### Kir6.1-Containing Endothelial K_ATP_ Channels in Conduit Vessels

**Figure 2.**
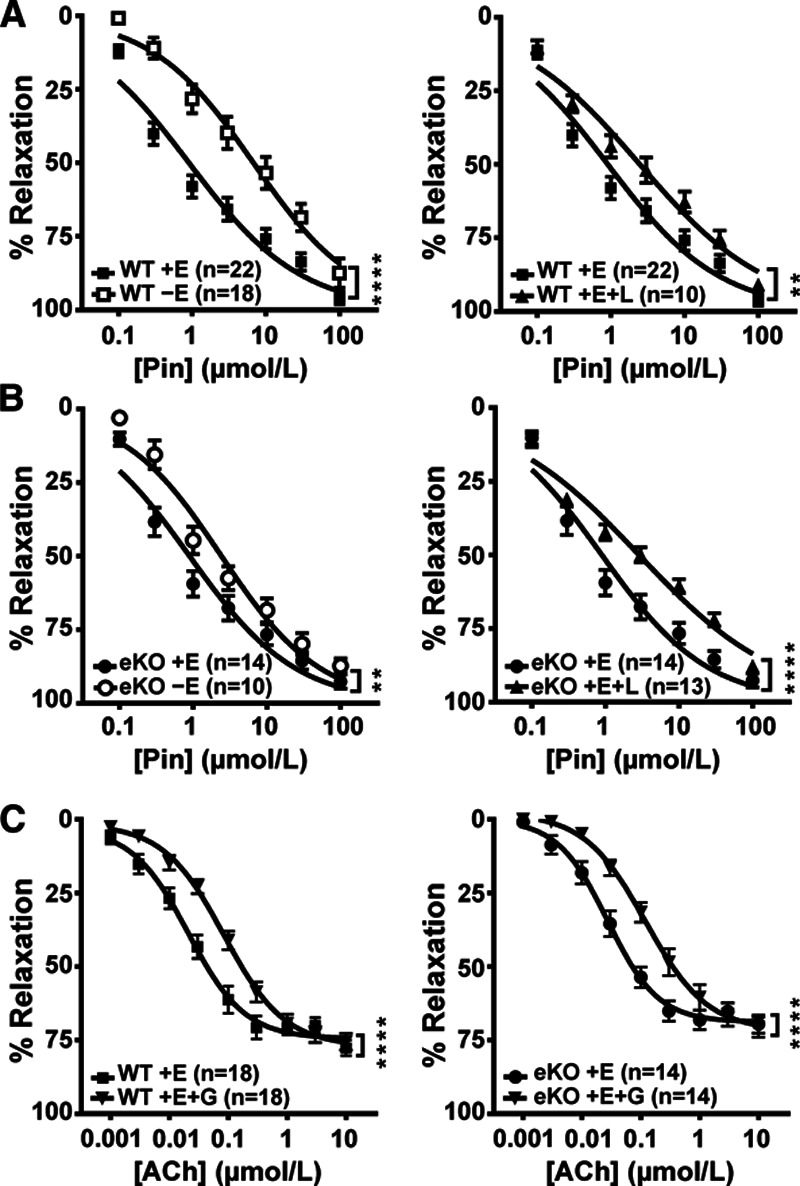
Effect of endothelium denudation, L-NAME, and glibenclamide on pinacidil- and acetylcholine-induced relaxations in a. Daortose-response curves for pinacidil-induced relaxation of aortic rings with intact (+E) or denuded (−E) endothelium from WT (**A**) and eKO (**B**) mice. Vessels were precontracted with 1 μmol/L phenylephrine. Endothelium-intact vessels were also challenged with 300 μmol/L L-NAME (L). **C**, Dose-response curves of acetylcholine (ACh)-induced relaxation in the presence and absence of 10 μmol/L glibenclamide (G) in WT (+E) and eKO (+E) mice. Data are shown as mean±SEM, **P<0.01, ****P<0.0001 compared with WT.

To investigate the effect of deleting Kir6.1 in the endothelium of conduit vessels, we compared the relaxation of endothelium intact (+E) and denuded (−E) aortic rings from 10- to 12-week-old wildtype and eKO mice with pinacidil (0.1–100 μmol/L). In wildtype mice, endothelial denudation shifted the pinacidil dose-response curve to the right (Figure 2A; *P*<0.0001). The application of the NO synthase inhibitor L-NAME (300 μmol/L) to endothelium-intact vessels also significantly shifted pinacidil-induced relaxation to the right (Figure 2A; *P*<0.01). In comparison, in eKO mice, removal of the endothelium had a much smaller but still significant effect on pinacidil-induced relaxation (Figure 2B; *P*<0.01). The application of L-NAME also significantly shifted the pinacidil-induced relaxation to the right (Figure 2B; *P*<0.0001). We also tested the effect of the K_ATP_ inhibitor glibenclamide (10 μmol/L) with a K_ATP_-independent, NO-dependent vessel relaxant, acetylcholine (Figure 2C). Glibenclamide significantly inhibited acetylcholine-induced relaxation in both wildtype (*P*<0.0001) and eKO mice (*P*<0.0001). The dose-response curves to pinacidil (EC_50_; WT: 1.29±0.40 µmol/L, eKO: 1.11±0.25 µmol/L, *P*>0.05) and acetylcholine (EC_50_; WT: 0.26±0.12 µmol/L, eKO: 0.45±0.15 µmol/L, *P*>0.05) between eKO and wildtype mice were not significantly different in endothelium-intact rings.

### Kir6.1-Containing Endothelial K_ATP_ Channels in Resistance Vessels

**Figure 3.**
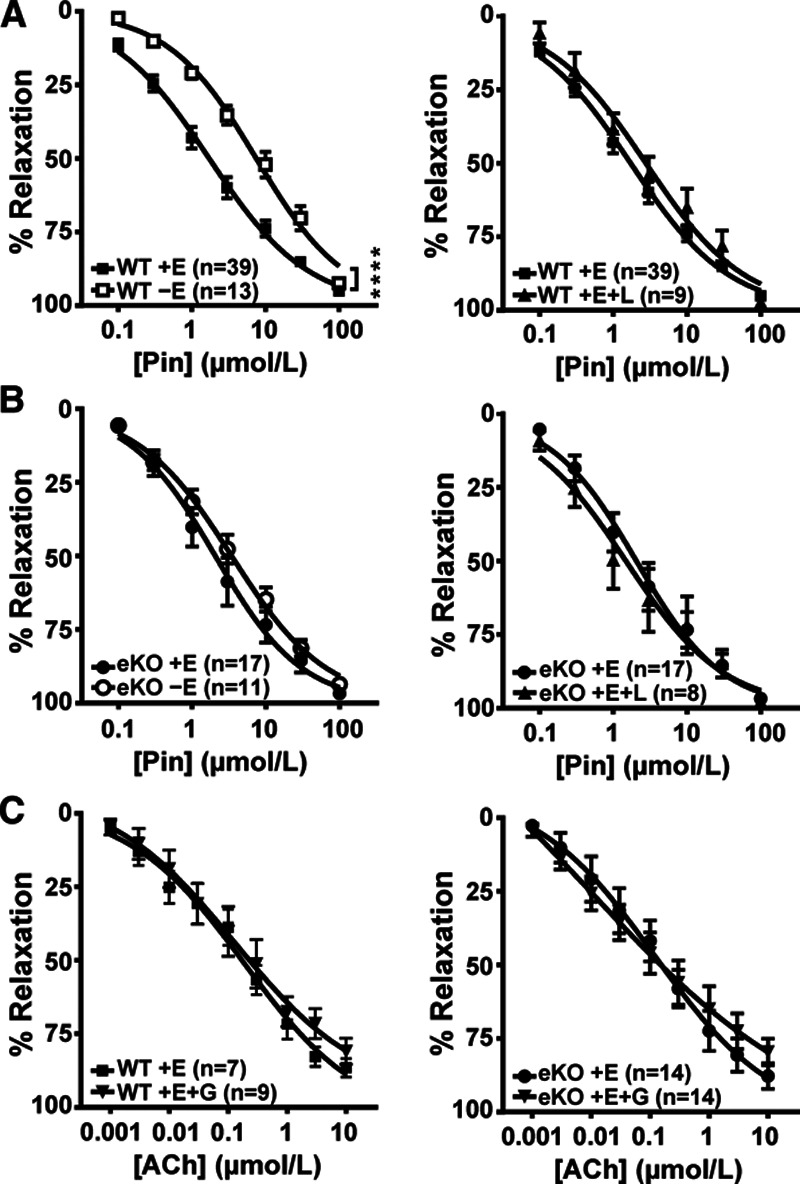
Effect of endothelium denudation, L-NAME and glibenclamide on pinacidil- and acetylcholine-induced relaxations in mesenteric arteries. Dose-response curves for pinacidil-induced relaxation of mesenteric arterial rings with intact (+E) or denuded (−E) endothelium from WT (**A**) and eKO (**B**) mice. Vessels were precontracted with 3 μmol/L phenylephrine. Endothelium-intact vessels were also challenged with 300 μmol/L L-NAME. **C**, Dose-response curves of acetylcholine (ACh)-induced relaxation in the presence and absence of 10 μmol/L glibenclamide (G) in WT (+E) and eKO (+E) mice. Data are shown as mean±SEM, ****P<0.0001 compared with WT.

To investigate the effect of endothelial Kir6.1 deletion in resistance vessels, we compared the relaxation of endothelium-intact and endothelium-denuded mesenteric artery rings from 10- to 12-week-old wildtype and eKO mice to pinacidil. Denudation of wildtype mesenteric rings shifted the pinacidil dose-response curve to the right (Figure 3A, *P*<0.0001). The application of L-NAME to wildtype endothelium-intact rings had no effect on the pinacidil dose-response curve (Figure 3A, *P*>0.05). This is likely due to the known actions of endothelium-derived hyperpolarization of VSM cells either through direct coupling or the release of mediators particularly in high resistance vessels to compensate for the lack of NO.^18–23^ In comparison, for eKO mice, removal of the endothelium had no effect in mesenteric rings (Figure 3B, *P*>0.05) and in endothelium-intact rings L-NAME had no effect on the pinacidil-induced relaxation (Figure 3B, *P*>0.05). Additionally, acetylcholine-induced relaxation was not affected by glibenclamide in either wildtype or eKO mice (Figure 3C, *P*>0.05). As in the aortic rings, there was no significant difference for the pinacidil-induced (EC_50_; WT 2.80±0.50 µmol/L, eKO 2.29±0.82 µmol/L, *P*>0.05) and acetylcholine-induced (EC_50_; WT 0.25±0.01 µmol/L, eKO 0.47±0.22 µmol/L, *P*>0.05) relaxations of endothelium-intact mesenteric rings in wildtype and eKO mice. These data support the presence of a Kir6.1-containing endothelial K_ATP_ channel in mesenteric arteries, the actions of which are not sensitive to NO inhibition.

### eKO Mice Develop More Pronounced Endothelial Dysfunction When Challenged With a High-Salt Diet but Not With a High-Fat Diet

**Figure 4.**
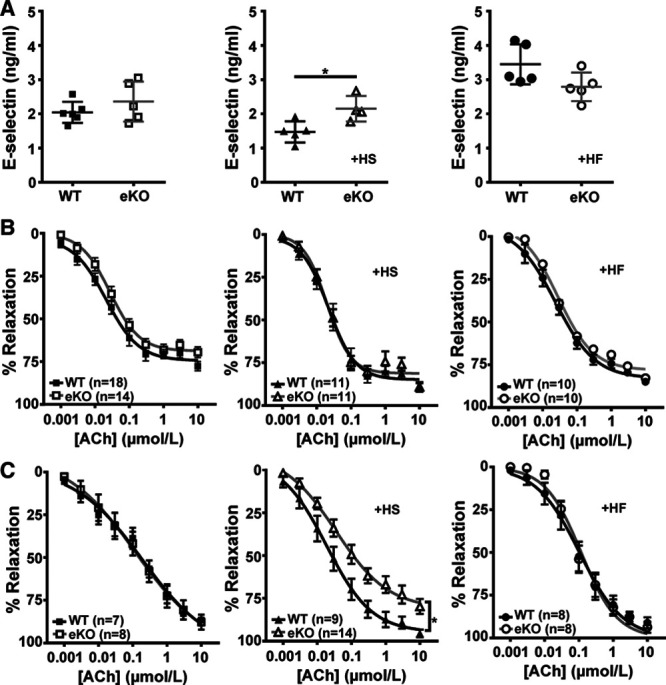
Effects of high salt and high fat diets on plasma E-selectin levels and acetylcholine (ACh)-induced relaxations in mouse aorta and mesenteric arteries. **A**, Mean plasma E-selectin levels in WT and eKO mice following sham, high salt (HS, 4 wk) and high fat diet (HF, 26 wk). **B**, Dose-response curves of ACh-induced relaxation of aortic rings from WT (+E) and eKO (+E) mice following sham, HS and HF diet. **C**, Dose-response curves of ACh-induced relaxation of mesenteric arterial rings from WT (+E) and eKO (+E) mice following sham, HS and HF diet. Vessels were precontracted with phenylephrine (sham and HS) or U46619 (HF). Data are shown as mean±SEM, each dot represents one animal, *P<0.05.

Endothelial dysfunction is often observed in disease states such as hypertension and obesity, we therefore investigated the role of Kir6.1-containing K_ATP_ channels in the functional regulation of blood flow when WT and eKO mice were on a HS or high fat (HF) diet for a prolonged period of time. Groups of WT and eKO mice were placed on either a sham (normal diet), HS (8% NaCI), or HF (42% fat) diet when they were 6- to 8-week-old for 4 (HS) and 26 (HF) weeks. Plasma E-selectin was used as a biomarker of endothelial dysfunction. Plasma E-selectin levels were similar in mice on the sham diet (Figure 4A, WT 2.04±0.13 ng/mL, eKO 2.36±0.26 ng/mL, *P*>0.05). However, in mice on a HS diet, plasma E-selectin levels were significantly higher in eKO compared with wildtype mice at 4 weeks indicating possible endothelial dysfunction in eKO mice (Figure 4A, WT 1.47±0.14 ng/mL, eKO 2.15±0.19 ng/mL, *P*<0.05). In contrast, in mice on a HF diet (26 weeks), although the mean plasma E-selectin levels were higher than mice on a sham diet, there was no significant difference between wildtype and eKO mice (Figure 4A, WT 3.45±0.26 ng/mL, eKO 2.79±0.19 ng/mL, *P*>0.05). We then compared the relaxation of the aorta from these mice to endothelium-dependent stimulation with acetylcholine; however, we did not see any difference between wildtype and eKO mice on all 3 diets (Figure 4B, Table; *P*>0.05). In addition, there was no difference in acetylcholine-induced relaxations in the mesenteric arteries of these mice on a sham or a HF diet (Figure 4C, Table; *P*>0.05). However, there was a clear rightward shift of the acetylcholine dose-response curve in eKO mice compared with wildtype mice fed a HS diet, (Figure 4C, Table; *P*<0.05). These data suggest endothelial dysfunction in eKO mice fed a HS diet occurring predominantly in resistance vessels but not with a HF challenge.

**Table.**
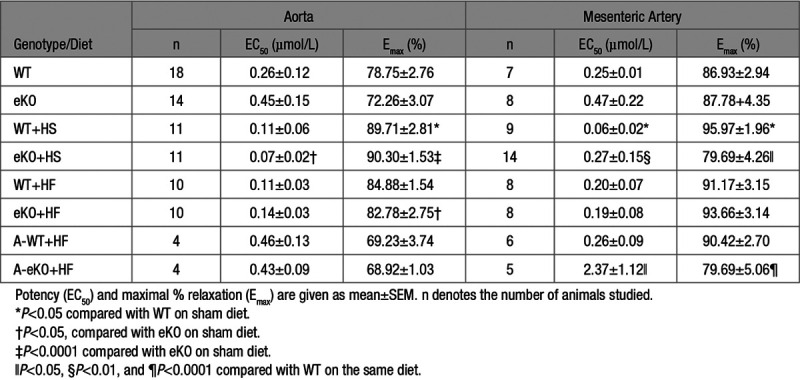
Effects of Sham, High Salt and High Fat Diets on Ach-Induced Relaxations in Mouse Aorta and Mesenteric Arteries

### eKO Mice on an Apolipoprotein E (ApoE) KO Background Unmasked More Pronounced Endothelial Dysfunction When Challenged With a High-Fat Diet

**Figure 5.**
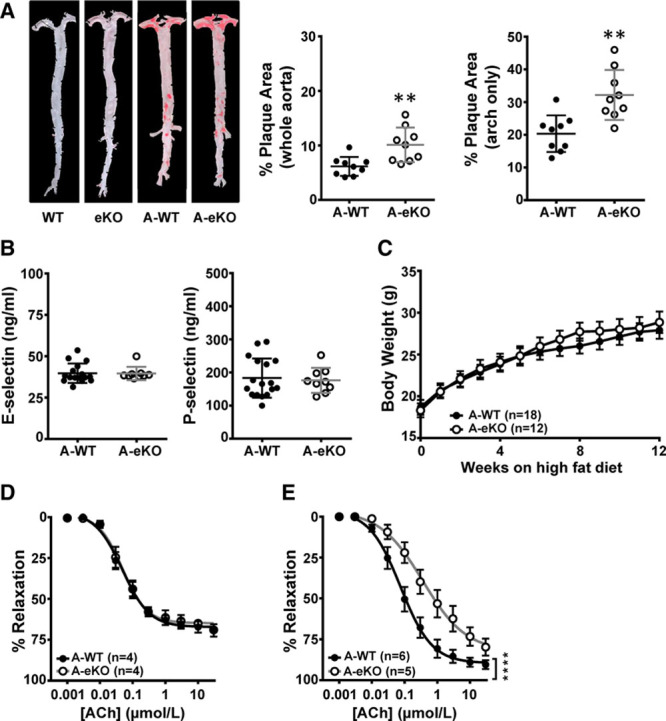
Effects of high fat diet in various murine strains. **A**, Representative images (left) and quantification (right) of Oil Red O-stained aortas. **B**, Mean plasma E-selectin and P-selectin levels in A-WT and A-eKO mice. **C**, Body weight development in A-WT and A-eKO mice receiving high-fat diet. **D**, Dose-response curves of acetylcholine (ACh)-induced relaxation of aortic rings from A-WT (+E) and A-eKO (+E) mice. **E**, Dose-response curves of ACh-induced relaxation of mesenteric arterial rings from A-WT (+E) and A-eKO (+E) mice. Vessels were precontracted with U46619. Data are shown as mean±SEM, each dot represents one animal, **P<0.01, ****P<0.0001.

To investigate the effects of HF consumption in eKO mice we crossed these mice with ApoE^−/−^ mice to generate A-eKO mice and their littermate controls (A-WT). ApoE^−/−^ mice are known to develop fatty deposits in the aorta in response to high-fat feeding. In atherogenesis, endothelial dysfunction contributes to the formation of atherosclerotic plaques on the arterial wall. A-eKO and A-WT mice were put on a HF diet when at 5 to 6 weeks of age for 12 weeks. We found that there was a significant increase in atherosclerotic plaque formation in aortas of A-eKO mice compared with A-WT mice (Figure 5A, A-WT 6.15±0.58%, A-KO 10.11±1.06%, *P*<0.01), with the highest plaque burden found in the aortic arch region (Figure 5A, A-WT 20.35±1.86%, A-KO 32.18±2.55%, *P*<0.01). Surprisingly, although there were elevated levels of plasma E-selectin and P-selectin compared with mice on a normal background, these were not significantly different between A-WT and A-eKO mice (Figure 5B, E-selectin, A-WT 39.69±1.47 ng/mL, A-eKO 39.61±1.33 ng/mL, *P*>0.05; P-selectin, A-WT 183.10±14.35 ng/mL, A-eKO 176.10±12.60 ng/mL, *P*>0.05). Both A-WT and A-eKO mice exhibit similar body weight development over the 12 weeks on an HF diet (Figure 5C, *P*>0.05). In addition, the acetylcholine dose-response curve was shifted to the right in A-eKO mice compared with A-WT mice in the mesenteric arteries (Figure 5E, Table, *P*<0.0001) but not in aortas (Figure 5D, Table, *P*>0.05). Taken together, these data suggest that endothelial Kir6.1-containing K_ATP_ channels may have a protective role in the vasculature against development of atherosclerosis.

## Discussion

In the present study, we have investigated the role of the endothelial K_ATP_ channel in vivo and in vitro under normal and pathological conditions in novel mouse strains that lack the pore-forming Kir6.1 subunit in vascular endothelium. The endothelial cells lining the luminal surface of the entire vasculature play an important role in regulating the vascular tone of the surrounding VSM cells.^24^ They do so by secreting an array of vasodilators and vasoconstrictors, such as NO, prostacyclin, and endothelin and also by direct propagation of endothelial cell hyperpolarization via myoendothelial gap junctions.^18–23,25^ Endothelial dysfunction caused by an imbalance of these vasodilators and vasoconstrictors is present in many pathological conditions, for example, it could promote the development of hypertension and is regarded as an early marker for predisposition to atherosclerosis.^26,27^

In our previous work, the smooth muscle–specific Kir6.1 KO mouse was found to be moderately hypertensive but not as hypertensive as the mouse with global deletion of Kir6.1.^2^ Given the ability of the endothelial K_ATP_ channel to modulate cytosolic Ca^2+^^5^ and thus potentially the release of vasoactive mediators, it raises the question of the contribution of the endothelial K_ATP_ channel to BP regulation. From our in vivo telemetry data, we found that there was no change in basal BP parameters in eKO mice compared with wildtype littermate controls. This is perhaps not surprising as the dose-response curve to the K_ATP_ opener pinacidil was not significantly different in eKO mice in both aortas and mesenteric arteries compared with WT mice. Likewise, responses to the endothelium-dependent vasodilator acetylcholine in eKO mice were unaffected under basal conditions in both aorta and mesenteric arteries. Finally, there was no evidence of cardiac hypertrophy in eKO mice compared with littermate controls. One significant change was a slower heart rate in the eKO mice, which might be indicative of a compensatory change via baroreflex to normalize BP.

Complementary to our previous work and that of others, we provide further evidence for the widespread distribution of K_ATP_ channels in vascular endothelium from pharmacological studies of pinacidil-induced relaxation in aortic and mesenteric artery rings. In both conduit and resistant vessels, responses to pinacidil were compromised when endothelium was removed, and this change was less or no longer occurred in eKO mice. Interestingly, the pinacidil-evoked vasorelaxation was higher in eKO mice compared with WT mice. It is unclear why this is but it could be because of an enhanced compensatory response from the smooth muscle. It is known that the relaxation evoked by acetylcholine in conduit vessels is endothelium-dependent and is mediated by nitro oxide synthesized endogenously by NO synthase (eNOS) from L-arginine.^25,28,29^ Organ bath studies on aorta suggest that the NO pathway might well be involved in K_ATP_ channel activity as addition of the eNOS inhibitor L-NAME had an attenuating effect on pinacidil-induced relaxations. Acetylcholine-induced relaxation was sensitive to the K_ATP_ blocker glibenclamide in both WT and eKO mice. Although speculative, this could be due to an enhanced blockade of VSM K_ATP_ channels by glibenclamide. Interestingly, mesenteric arteries were unaffected by either L-NAME or glibenclamide. This may simply reflect the fact that in these resistance arteries, other vasodilatory mechanisms involving EDHF and a direct propagation of endothelial cell hyperpolarization to smooth muscle play a more prominent role.^18–23,30^

Although we did not see a basal BP phenotype in eKO mice, we found that when the 2 groups of mice were made hypertensive by challenging them with a HS diet both with and without L-NAME administration, BP was significantly higher in eKO mice. Furthermore, mice lacking Kir6.1 in the endothelium had a faster initial increase in BP after introduction of the diet which was sustained over the 4-week period of the diet suggesting that under hypertensive stress endothelial K_ATP_ channels may perform a compensatory role by offsetting some of the rise in BP. This could be through a direct release of vasoactive mediators or via endothelial KATP-mediated changes in endothelial cell membrane potential that is directly conducted to the smooth cells through myoendothelial gap junctions to produce vasorelaxation.^18–23^ However, the channel may more generally protect the endothelium against endothelial dysfunction such as would occur with the development of hypertension.^31,32^ In support, eKO mice also have elevated plasma E-selectin levels and diminished acetylcholine relaxation responses in mesenteric arteries. In WT mice, E-selectin levels are actually lower after a HS challenge compared with basal levels suggesting that K_ATP_ channels may protect against endothelial dysfunction by reducing E-selectin levels rather than preventing an increase. Elevated plasma E-selectin levels may also be indicative of an inflamed endothelium, and it is possible that endothelial K_ATP_ channels modulate the response to HS-mediated inflammation.^33^ Furthermore, the heart rate drops as BP rises in these mice, probably related to activation of the baroreceptor reflex without time to reset to a new set-point. Interestingly, heart rate was lower in eKO mice even under basal conditions before challenge, significantly so during the day. While as previously discussed this may result from changes in the baroreceptor reflex, cardiac ECs also express and release a range of auto- and paracrine mediators such as NO, PGI_2_, and endothelin which control important cardiac functions namely contractility and rhythm.^34^ It may be that K_ATP_ channels are also expressed in the endocardium as well as vascular ECs and contribute to the modification of cardiac function.

We also challenged the mice with a HF diet and this intervention can lead to increased body weight, hyperglycemia, hyperinsulinemia, cardiac dysfunction, and hypertension.^35^ Endothelial dysfunction is often observed in obese animals and a reduction in endothelial NO bioavailability is often the leading mechanism.^36–38^ Endothelial dysfunction is closely associated with the development of atherosclerosis, with obesity and insulin resistance being the major risk factors.^39^ In mice fed on a HF diet over a course of 26 weeks, we observed no difference in the acetylcholine-induced relaxations in both aortas and mesenteric arteries in eKO mice compared with littermate controls. Although the E-selectin levels were higher than in mice on a sham diet, there was no difference between WT and eKO mice. In addition, there was no evidence of atherosclerotic plaque formation in both WT and eKO mice. This is likely due to the relatively high natural resistance of mice to atherosclerosis.^40^ Therefore, to unmask the potential role of endothelial K_ATP_ in response to a HF diet, we crossed the eKO mice onto the Apolipoprotein E (ApoE) KO background to generate A-eKO and A-WT mice. ApoE^−/−^ mice have long been used as an animal model to investigate the progression of atherosclerosis and they develop atherosclerotic lesions resembling fatty streaks.^40,41^ Obvious plaque formation was seen in both control and A-eKO mice at 12 weeks on HF with the percentage area of atherosclerotic plaques significantly higher in the A-eKO mice, with most of the plaques formed around the aortic arch region. This is not surprising, given that in human vasculature, atherosclerotic lesions are often found at regions with altered wall shear stress such as bends, bifurcations and T-junctions.^42^ While plasma E-selectin and P-selectin levels were a lot higher in mice on the ApoE^−/−^ background, this was not different between A-eKO mice and A-WT mice. However, there was an attenuated acetylcholine relaxation in mesenteric arteries of the A-eKO mice, which suggests some endothelial dysfunction.

There are some limitations of our study. The tie2 cre recombinase driver line is widely used for endothelial deletion. However, it should be borne in mind that it also deletes in hemopoietic cells and K_ATP_ channels have been described in monocytes though the subunit composition is not clear.^43–45^ Thus, it is possible that inflammatory responses might be modulated. However, it is clear from the ex-vivo studies that there is intrinsic endothelial dysfunction. Some of the pharmacological agents have limitations; pinacidil has been reported to lead to vasorelaxation in a K_ATP_ independent manner and glibenclamide to block CFTR.^46,47^ However, our prior work indicates the predominant effect is via K_ATP_ channels and we use a concentration of glibenclamide (10 µmol/L) that leads to little inhibition of CFTR.^2,5,46,47^

In conclusion, the findings in our study support the existence of an endothelial Kir6.1-containing K_ATP_ channel in both mouse aortas and mesenteric arteries. They seem to have minimal contribution under basal physiological conditions; however, upon pathological challenge, they (1) contribute to the regulation of BP when challenged with a hypertensive stimulus and (2) play a protective role in preventing excessive atherosclerotic plaque formation.

## Perspectives

We have shown that K_ATP_ channels expressed in the endothelium protect against the development of hypertension and atherosclerosis in response to increased dietary salt and fat intake. This reveals new roles for these channels in ameliorating cardiovascular pathology.

## Acknowledgments

We are grateful to Dr Amie Moyes for technical assistance and Professor Adrian Hobbs for kindly providing the Tie2Cre+ and ApoE^−/−^ mice. Y. Li, Q. Aziz and A. Tinker conceived the study. Y. Li, Q. Aziz, N. Anderson, and L. Ojake performed the experiments and analyzed the data. Y. Li, Q. Aziz, and A. Tinker prepared the manuscript.

## Sources of Funding

This research was supported by the British Heart Foundation (RG/15/15/31742) and was facilitated by the NIHR Cardiovascular Biomedical Research Centre at Barts.

## Disclosures

None.

## Supplementary Material



## Novelty and Significance

### What Is New?

Blood pressure has been shown to be regulated by K_ATP_ channels in vascular smooth muscle. Here, we show the role K_ATP_ channels in vascular endothelium play in the pathogenesis of hypertension and atherosclerosis.

### What Is Relevant?

In this study, we show that endothelial K_ATP_ channels do not contribute to basal blood pressure modulation. They do, however, play an important role in protecting against the development of hypertension and atherosclerosis when subjected to genetic, pharmacological and dietary challenges.

### Summary.

Endothelial K_ATP_ channels protect against the development of pathological changes in response to environmental stress/stimuli.
