# Supplementary material for: Endothelial ATP-Sensitive Potassium Channel Protects Against the Development of Hypertension and Atherosclerosis
Source: Hypertension. 2020 Jul 13;76(3):776–84. doi: 10.1161/HYPERTENSIONAHA.120.15355 (PMC7418932; doi:10.1161/HYPERTENSIONAHA.120.15355)
Supplement: Supplementary file 1 [file hyp-76-0776-s001.pdf]

DATA SUPPLEMENT

The endothelial ATP-sensitive potassium channel protects against the development of hypertension and atherosclerosis

Yiwen Li PhD, Qadeer Aziz PhD, Naomi Anderson MSc, Leona Ojake BSc, Andrew Tinker MB BS FRCP PhD [#]

The Heart Centre, William Harvey Research Institute, Barts and the London School of Medicine and Dentistry, Queen Mary University of London, Charterhouse Square, London, EC1M 6BQ, UK.

# Corresponding Author; a.tinker@qmul.ac.uk, Tel: 02078825783 Fax: 02078823408

Generation of the endothelium-specific Kir6.1 Mouse Strains

Kir6.1(+/flx) mice were generated in our lab in collaboration with Genoway (Lyon, France; project number genOway/EV/TIN1- Kcnj8 070206), a detailed method has been described previously (*1*). Endothelium-specific Kir6.1 KO mice were generated by crossing endothelium Tie2 promoter-driven cre-transgenic mice (tie2cre) with Kir6.1 homozygous floxed (Kir6.1(flx/flx)) mice. A further cross of the offspring resulted in genotypes of tie2cre+Kir6.1(flx/flx) (eKO) and littermate controls (referred to as wildtype (WT) mice). Tie2cre+Kir6.1 mice were then crossed with apolipoprotein E knockout ($ApoE^{-/-}$) mice to generate $ApoE^{-/-}$tie2cre+Kir6.1(flx/flx) (A-eKO) and $ApoE^{-/-}$Tie2cre+Kir6.1(WT/WT) (A-WT) littermates. Tie2cre mice and $ApoE^{-/-}$ mice were kindly provided by Professor Adrian Hobbs.

Genotyping

DNA was extracted from mouse ear biopsies by proteinase K digestion. The presence of the Kir6.1 floxed allele was confirmed using the following primer set: sense 5'-ACTAGCACCTCTATCCCCAGCTCCTACC-3' and antisense 5'-CCGCCCCTCCCTCTGAACCTATATC-3' yielding WT bands of 458 bp and floxed allele bands of 584 bp. PCR cycling conditions were as follows; denaturation at 94 ºC for 2 minutes, 35 cycles of 94 ºC for 30 secs, 63 ºC for 30 secs, 68 ºC for 1 minute, extension at 68 ºC for 8 minutes. The presence of the cre recombinase gene in the Kir6.1 endothelium-specific knock-out lines was determined using the following primer set: sense 5'-CCCTGTGCTCAGACAGAAATGAG-3' and antisense 5'-CGCATAACCAGTGAAACAGCATTGC -3' yielding a band of 512 bp in cre positive mice and no band in cre negative mice. PCR cycling conditions were; denaturation at 95 ºC for 10 minutes, 40 cycles of 95 ºC for 30 seconds, 58 ºC for 1 minute, 72 ºC for 1 minute, extension at 72 ºC for 10 minutes. The presence of the ApoE allele was confirmed using the following primer set; sense 5'-GCCTAGCCGAGGGAGAGCCG-3', antisense WT 5'- TGTGACTTGGGAGCTCTGCAGC-3', antisense mutant 5'-GCCGCCCCGACTGCATCT-3', yielding a WT bands of 150 bp and a knockout band of 250 bp. PCR cycling conditions were denaturation for 3 minutes at 94 ºC, 35 cycles of 94 ºC for 30 secs, 68 ºC for 40 secs, 72 ºC for 1 minute, and extension for 2 minutes at 72 ºC. All PCRs were performed using BioMix™ Red (Bioline BIO-25006).

Organ bath and wire myography experiments

Mice were killed by cervical dislocation. The aorta and mesenteric bed were dissected and placed in freshly-made Krebs-bicarbonate buffer composed of (mmol/L): NaCl 119, KCl 4.7, $CaCl_2$ 2.5, $MgSO_4$ 1.2, $NaHCO_3$ 25, KH_2PO_4 1.2, and glucose 5.5, maintained at room temperature and gassed with 95 % CO_2/5 % O_2. The aorta and 2nd order mesenteric arteries (diameter ≈ 150 μM) were cleaned of connective tissue and blood, and then cut into rings (length ≈ 2 mm long). Aortic and mesenteric rings were mounted into a four-chamber Mulvany-Halpern myograph (model 620M, DMT Denmark) using pins or jaws with 25 μm tungsten wires (Goodfellow, UK). Both sets of arteries were allowed to equilibrate for 60 mins with washes every 15 mins with Krebs-bicarbonate buffer before the start of the experiment. During the equilibration period, aortic rings were given a resting tension of 3 mN, and the mesenteric arterial rings were normalized to an inner diameter corresponding to a maximum pressure of 13.3 kPa (100 mmHg). After equilibration, aortic rings were exercised twice with 48 mmol/L KCl or 1 μmol/L thromboxane-A2 mimetic U46619 (only in high fat diet mice)

and were allowed to re-equilibrate over a 30 min period with washes every 10 mins to remove the KCl. 1 µmol/L phenylephrine (PE) (aorta) or 3 µmol/L PE (mesenteric) was used to induce the sub-maximal contraction and the concentration-dependent relaxation response to pinacidil or acetylcholine (ACh) in the absence and presence of 10 µmol/L glibenclamide (Glib) and 300 µmol/L Nω-Nitro-L-arginine methyl ester hydrochloride (L-NAME) was recorded. In mice challenged with the high fat diet (42% fat, Envigo TD88137), U46619 was added cumulatively until it induced contractions 50 % of the maximal U46619 response. In some preparations, the vessels were denuded of their endothelium. In the aortic rings, this was done by insertion of curved metal forceps into the lumen and gently rolling the vessel along the fingertip. For mesenteric rings, a human hair was inserted into the lumen to remove the endothelium. Endothelial denudation was confirmed by the inability of a PE-contracted ring to relax following 10 µmmol/L ACh addition. Data were captured using Powerlab and LabChart version 8 (AD Instruments, UK). Pharmacological agents were purchased from Sigma-Aldrich (UK) and were dissolved in Krebs-bicarbonate buffer, except pinacidil/glibenclamide (DMSO) and U46619 (ethanol).

Tail cuff blood pressure measurements

Blood pressure measurements were made non-invasively using the tail cuff method (CODA, Kent Scientific, Torrington, VT, USA). A specialized volume pressure recording (VPR) sensor was placed over the animal's tail and blood volume changes were measured. Mice were restrained in specific holders and artificially heated to maintain normal BP. Mice were trained for five consecutive days before baseline readings were recorded. Subsequent recordings when the mice were on a high salt diet were then carried out weekly at the same hour over a four-week periods. All measurements were obtained by an investigator blinded to the identity of the experimental groups.

Analysis of Atherosclerotic Plaques

The entire aorta (from the aortic root to the iliac bifurcation) was dissected free of adipose tissues, fixed in 10% formalin for 24 hours, prepared en face and stained with Oil Red O (Sigma-Aldrich, UK). Images were captured using a Canon 500D digital camera. The aortic lesion area and total aortic area were calculated using Image J software.

ELISA

Blood was collected from anesthetised mice via cardiac puncture using syringes coated with heparin. Plasma was immediately separated and stored at -20°C until analysis. For a quantitative analysis of endothelial function, levels of E-selectin and P-selectin were measured using commercially available ELISA kits according to the manufacturer's instructions (Abcam, ab171182, R&D systems DY575 and DY737). Optical density absorbance was measured with a Victor2 1420 Multilabel Counter (Perkin Elmer).

Statistical analysis

Investigators were blinded to the genotype of the animals for analysis and quantification of the data. All data are presented as mean±SEM and were analysed using Microsoft Excel and GraphPad Prism. In the organ bath and wire myography

experiments, tests of significance between dose-response curves were conducted using two-way ANOVA with repeated measures followed by Bonferroni's multiple comparisons test. Unpaired Student’s t-test and ANOVA were used to compare means where appropriate. N values indicate the number of animals in each group. $P \leq 0.05$ was taken to be significant.

References

1. Q. Aziz *et al.*, The ATP-sensitive potassium channel subunit, Kir6.1, in vascular smooth muscle plays a major role in blood pressure control. *Hypertension* **64**, 523-529 (2014).
