# Supplementary figures and images for: Endothelial ATP-Sensitive Potassium Channel Protects Against the Development of Hypertension and Atherosclerosis
Source: Hypertension. 2020 Jul 13;76(3):776–84. doi: 10.1161/HYPERTENSIONAHA.120.15355 (PMC7418932; doi:10.1161/HYPERTENSIONAHA.120.15355)

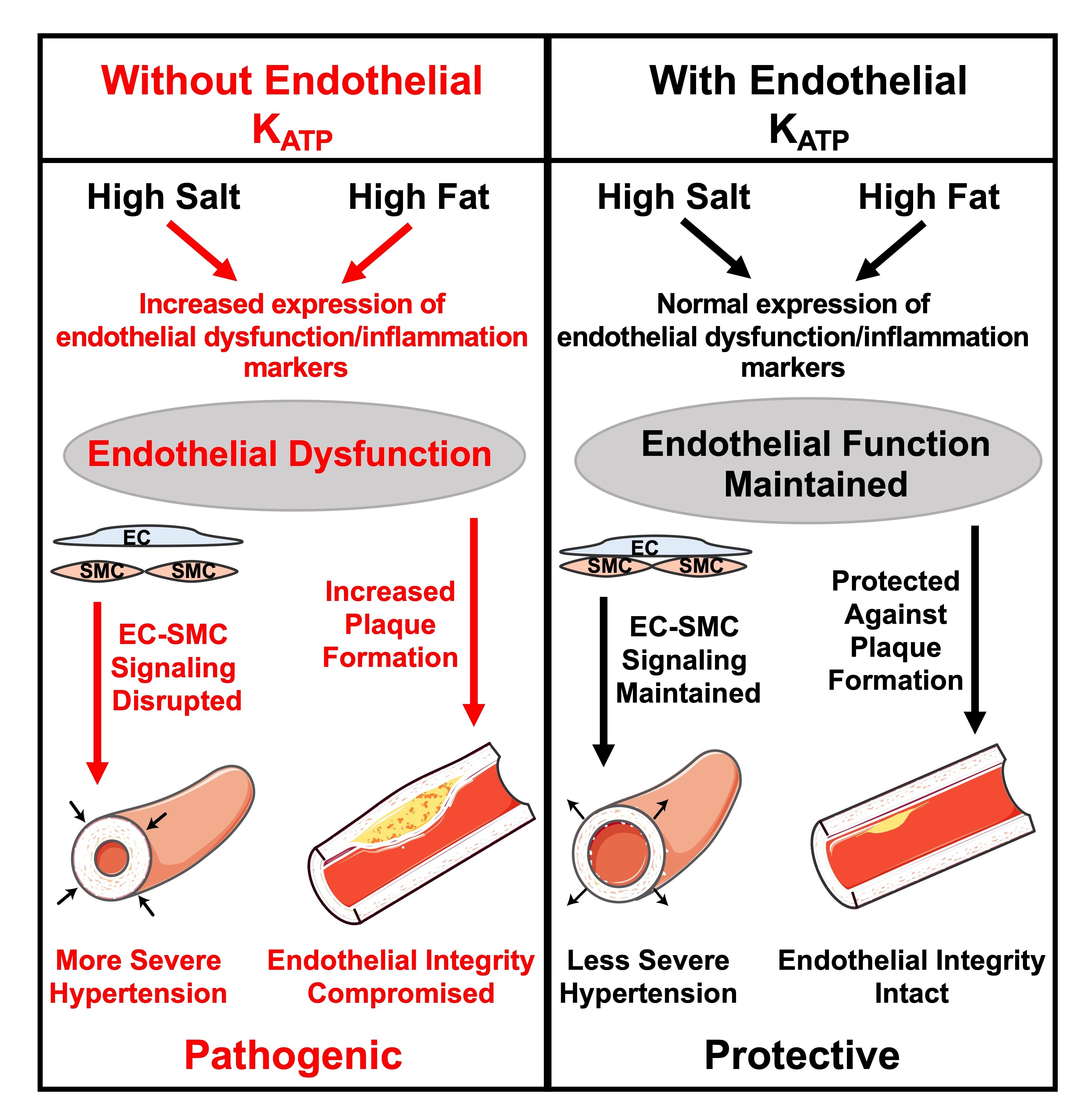

Supplement: Supplementary file 2 [file hyp-76-0776-s002.jpg]
