# Supplementary material for: Endothelial ATP-Sensitive Potassium Channel Protects Against the Development of Hypertension and Atherosclerosis
Source: Hypertension. 2020 Jul 13;76(3):776–84. doi: 10.1161/HYPERTENSIONAHA.120.15355 (PMC7418932; doi:10.1161/HYPERTENSIONAHA.120.15355)
Supplement: Supplementary file 3 [file hyp-76-0776-s003.pdf]

*** Short In Vivo Checklist**
AHA - Preclinical Animal Testing: Prevention of bias is important for experimental cardiovascular research. ***This short checklist must be completed, and the answers should be clearly presented in the manuscript as well.*** *The checklist will be used by reviewers and editors but will not be published. If a revision is requested, you will be required to complete at revision submission a more detailed checklist that will be published with the accepted article.*

This study involves animals:
Yes

Animals

Species, age, sex, strains, and sources of animals are described:	Yes

Randomization

Randomization and allocation concealment were performed:	Yes

Blinding

Blinding was performed:	Yes

Inclusions and Exclusions (a)

Specific criteria for inclusions and exclusions are specified:	N/A

Inclusions and Exclusions (b)

Criteria for inclusions and exclusions were set before the study:	N/A

Reporting of Excluded Animals

All animals excluded after the randomization are reported:	N/A

Statistical Methods

Statistical Methods are described:	Yes

Date completed: 06/06/2020 06:55:10
User pid: 208724
